# How to crack a SMILES: automatic crosschecked chemical structure resolution across multiple services using MoleculeResolver

**DOI:** 10.1186/s13321-025-01064-7

**Published:** 2025-08-04

**Authors:** Simon Müller

**Affiliations:** Institute of Thermal Separation Processes, Hamburg University of Technology, Eißendorfer Straße 38, 21073 Hamburg, Germany

**Keywords:** Chemical structure retrieval, Identifier, SMILES, ML, QSPR, Python, MoleculeResolver

## Abstract

**Abstract:**

Accurate chemical structure resolution from textual identifiers such as names and CAS RN® is critical for computational modeling in chemistry and related fields. This paper introduces MoleculeResolver, an automated, robust Python-based tool designed to address inconsistencies and inaccuracies commonly encountered when converting chemical identifiers to canonical SMILES strings. MoleculeResolver systematically crosschecks structures retrieved from multiple reputable chemical databases, implements rigorous identifier plausibility checks, standardizes molecular structures, and intelligently selects the most accurate representation based on a unique resolution algorithm.

**Scientific contribution:**

Benchmarks across diverse datasets confirm that MoleculeResolver significantly enhances precision, recall, and overall reliability compared to traditional single-source methods, proving its utility as a valuable resource for chemists, data scientists, and researchers engaged in high-quality molecular data analysis and predictive model development.

**Graphical Abstract:**

**Figure:**
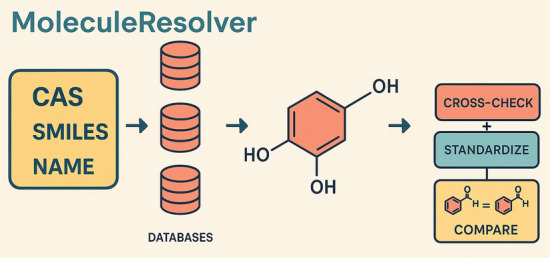

## Introduction

The future of data-driven molecular models hinges on a critical challenge: the fast, accurate and consistent machine-readable representation of chemical structures [1]. Inconsistent identifiers, erroneous metadata, and ambiguous structural representations have emerged as silent barriers to progress in computational modeling and machine learning [2–6]. Particularly in solvent screening, drug discovery, predictive thermodynamic or thermophysical properties, molecular modeling can help accelerate research, reduce costs, and minimize in vivo testing. This is especially true for the case where the component in question is explosive, toxic, hard to measure, or a large number of components are to be tested in order to find the most suitable one for the task.

ML (Machine Leaning), QSPR (Quantitative structure–property relationship), Group contribution (GC) approaches are examples of models to correlate a target property with the chemical structure [7–9]. However, the accuracy of these models depends heavily on the quality of the input data [10]. This includes the measurements itself [11], the accuracy of the metadata and especially the correctness of the chemical structures [3, 4, 12, 13].

A substantial amount of data for target properties (to be modeled) exists only in printed form, while other datasets are available digitally but lack associated structural information. In some cases, conflicting identifiers are present across sources. MoleculeResolver was developed to address the need for efficiently annotating large volumes of chemical identifiers with corresponding structural representations. In this regard, it shares similarities with the workflow introduced by Gadaleta et al. [12] for KNIME.

This work presents the key challenges associated with obtaining reliable chemical structure data, describes the methods implemented to address these challenges, and evaluates the performance of MoleculeResolver using various reference identifier sets. The package is available via pip, and the source code can be accessed through the corresponding GitHub repository [14]. The version used in this study is 0.3.4, as released on GitHub. Additionally, the source code is included in the Supporting Information.

### Challenges

**Fig. 1:**
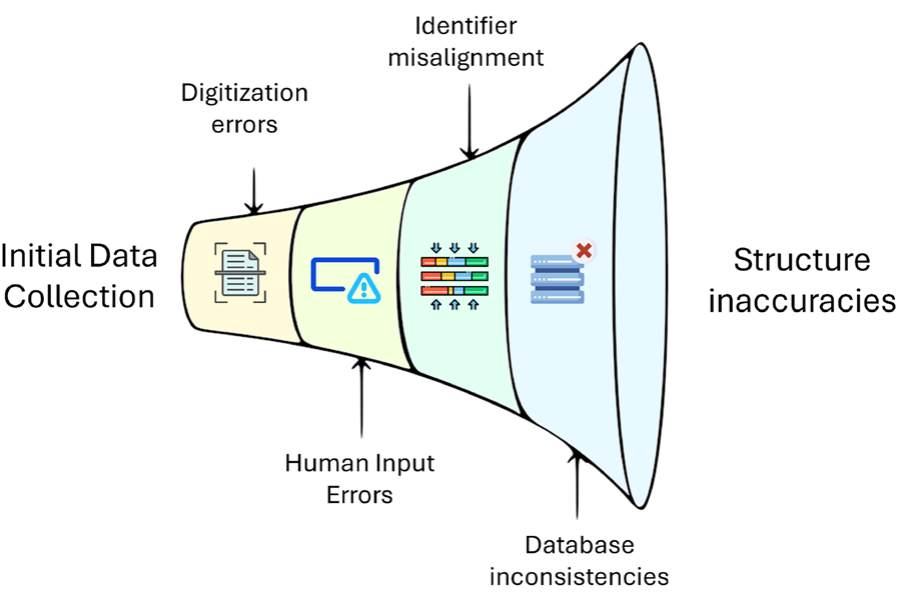
Accumulation of errors for structures

As illustrated in Fig. 1, errors in structures can originate from a variety of sources, including human errors [1]. The digitization of primary or compiled sources often depends on optical character recognition (OCR) technology, which may misinterpret certain characters. This can lead to identifiers that are unresolvable by any of the available services [15]. Even minor inconsistencies in data can accumulate across large datasets, posing challenges to model accuracy and reliability.

Although primary sources are generally regarded as reliable, they may still contain errors. Additionally, mistakes can be introduced during the manual transcription or compilation of identifiers. Further complications arise when multiple identifiers for a substance (e.g., chemical names, CAS RN®, etc.) are available, as these may not consistently map to the same chemical structure, thereby compounding the problem. Discrepancies also become evident when structures are retrieved across different services. Moreover, the inclusion or omission of stereoisomeric information can vary between services.

Such inconsistencies are particularly evident with non-systematic names, where retrieval services frequently diverge on the resulting structures, complicating reliable data acquisition [4, 16–18].

CAS RN®, although widely used as chemical identifiers, present several challenges that affect their consistency and reliability. Incorrect assignments may occur in public database services due to errors such as conflating parent and salt forms of a CAS RN® or misinterpreting stereochemistry from low-quality depictions [19, 20]. Inconsistencies can also arise when the chemical structures provided in databases do not correspond to those associated with the CAS RN® in the official registry.

In general, a single chemical structure may sometimes be associated with multiple CAS RN® or other unique identifiers, often as a result of discrepancies between regulatory inventories. At the same time, differing standardization protocols across services can lead to the same CAS RN® or identifier being linked to structurally divergent representations or alternate forms of the same compound [13, 15, 21, 22].

### Addressing the challenges

It is not feasible to resolve all underlying errors, as the ground truth is often unknown. Nevertheless, MoleculeResolver incorporates several measures aimed at improving the quality of retrieved chemical structures:

- **Identifier plausibility check**: For most identifier types (e.g., CAS RN®, InChI, InChIKey, SMILES), basic plausibility is assessed using regular expressions and, where applicable, validation algorithms. Furthermore, for identifiers such as InChI and SMILES, RDKit [23] is used to test whether the input can be used to generate a valid structure.
- **Crosschecking across multiple services**: By default, up to nine services are queried for the same identifier to enable cross-validation and identify the most likely correct structure (see “Current services” section for details). Assuming independent curation among services, the likelihood of identical errors occurring across all sources is reduced.
- **Multiple identifier search**: Structures can be retrieved using a list of identifiers associated with the same compound, such as synonyms or alternative names.
- **Standardization and canonicalization**: Before comparing structures from different sources, all retrieved representations are normalized and canonicalized to enable more reliable structural comparison. Canonicalization in the context of this work refers to generating the canonical SMILES as calculated by RDKit [23] to compare the equality of structures.
- **Selection of representative structure**: An algorithm has been developed to select a representative structure based on outcomes from several tens of thousands of structure queries. This approach is designed to resolve discrepancies when services return conflicting results.

### Similar tools

Besides the Python modules and web services specifically developed for each of the platforms listed in Table 1, several related tools have also been introduced:

StructRecon [2] is designed to retrieve comprehensive knowledge graphs for specific identifiers from multiple offline databases, focusing on broader contextual information rather than solely on structural resolution.

Another related Python module, developed in parallel with MoleculeResolver, was pura [24], which retrieves chemical structures from names. While pura shares some functionality with MoleculeResolver, active development has ceased following the integration of its lead maintainer into the MoleculeResolver team.

A separate application, Chemical-Resolver [25], offers a graphical user interface (GUI) and has incorporated MoleculeResolver as an optional backend for structure resolution.

## Methods

### Current services

Currently, the services listed in Table 1 are employed to retrieve the most appropriate chemical structure for a given identifier. This list may be revised in the future to enhance performance by incorporating additional services or removing existing ones.

Among these, OPSIN [26, 27] is distinct in that it uses rule-based parsing to convert chemical names into structures. In contrast, the remaining services rely on querying databases using the provided identifiers.

MoleculeResolver does not implement the full range of configuration options available in each underlying service. Instead, it prioritizes retrieving structures based on identifiers with a balance between accuracy and computational efficiency.

**Table 1:** Current services and identifiers

Service	Name	CAS RN®	Formula	SMILES	InChI	InChIKey	CID	Batch Search	Repositories
CAS Common Chemistry$^{\textrm{TM}}$[28]	$\checkmark$	$\checkmark$	$\times$	$\checkmark$	$\checkmark$	$\times$	$\times$	$\times$	
ChEBI [29]	$\checkmark$	$\checkmark$	$\checkmark$	$\checkmark$	$\checkmark$	$\checkmark$	$\times$	$\times$	
Chemeo [30]	$\checkmark$	$\checkmark$	$\times$	$\checkmark$	$\checkmark$	$\checkmark$	$\times$	$\times$	
CIR [31]	$\checkmark$	$\checkmark$	$\checkmark$	$\checkmark$	$\checkmark$	$\checkmark$	$\times$	$\times$	[32]
CompTox [33, 34]	$\checkmark$	$\checkmark$	$\times$	$\times$	$\times$	$\checkmark$	$\times$	$\checkmark$	
CTS [35]	($\checkmark$)	$\checkmark$	$\times$	$\checkmark$	$\times$	$\times$	$\times$	$\times$	
NIST Webbook [36]	$\checkmark$	$\checkmark$	$\checkmark$	$\checkmark$	$\times$	$\times$	$\times$	$\times$	[37]
OPSIN [26, 27]	$\checkmark$	$\times$	$\times$	$\times$	$\times$	$\times$	$\times$	$\checkmark$	[38, 39]
PubChem [40]	$\checkmark$	$\checkmark$	$\checkmark$	$\checkmark$	$\checkmark$	$\checkmark$	$\checkmark$	$\checkmark$	[41]
SRS [42]	$\checkmark$	$\checkmark$	$\times$	$\times$	$\times$	$\times$	$\times$	$\checkmark$	

ChemSpider was not used directly, as it is already integrated within the CIR service [43–45]. ChemIDplus and the Drug Information Portal were retired in 2022 [46].

While CTS supports name-based searches, extended testing showed that fewer than 5% of names were successfully resolved. As a result, name-based queries are no longer performed using this service.

### The crosschecking algorithm

When multiple structures are retrieved for a single identifier, the following algorithm is applied to select the most appropriate candidate using character-wise string comparison for the different SMILES:

- The algorithm first identifies SMILES strings that appear with a frequency meeting a predefined crosscheck threshold and selects the most frequently occurring one, i.e., the structure agreed upon by the majority of services.
- If ambiguity remains, the SMILES strings of all candidates are converted to their non-isomeric forms. If these representations are identical, the algorithm selects the more detailed (isomeric) version.
- If ambiguity still remains and the structure was retrieved via a name, the structure returned by OPSIN [27] is selected.
- If further ambiguity persists, internal consistency checks are performed. The retrieved names are re-evaluated using OPSIN [27] to verify whether they correspond to the same structure. If one structure is found to be consistent, it is selected.
- If no unique candidate can be determined through the above steps, the algorithm selects the first structure in lexicographical order to ensure deterministic output. A warning is issued if configured to do so.

### Additional features

- **Heuristic identifier expansion or correction**: For chemical names and CAS RN®, functions are provided to apply heuristic rules that address common formatting inconsistencies and frequent typographical errors found in published data. In the case of CAS RN®, the algorithm attempts substitutions with numerically adjacent keys on a standard keyboard layout to identify a valid alternative, continuing until a syntactically valid CAS RN® is obtained.
- **Parallelized search**: For large sets of identifiers, structure retrieval can be parallelized, typically resulting in a speedup of approximately fivefold.
- **Cached retrieval**: Retrieved structures are stored in a local SQLite database. Subsequent queries for the same identifier and service are then resolved from cache, enabling significantly faster retrieval without any requests needed to the services. An expiry date can be specified to delete old entries or project specific cache databases can be setup instead of the universal one for the package.
- **Expected structure type**: For each identifier, users can optionally specify the expected type of structure (e.g., neutral compound, salt, ion, or mixture). This information can help refine the search and improve result relevance when such classification is known in advance.

## Structure handling

All structures are standardized and canonicalized prior to being compared or returned by MoleculeResolver.

### Standardization and canonicalization

Standardization is essential due to the variability in how chemical structures are represented, the presence of errors and inconsistencies in databases, and the differing conventions used across data sources. Given the scale of modern chemical databases, manual curation and standardization are not feasible, necessitating automated approaches.

Structure standardization refers to the process of converting chemical representations into a consistent and unambiguous format. This is critical for accurate data analysis, structural comparison, and integration across systems. It ensures that identical molecules are treated equivalently, regardless of differences in their original input format or source [47].

This process typically involves multiple steps, all of which are fully configurable within MoleculeResolver. The steps implemented in MoleculeResolver use the methods implemented in RDKit [23], which itself are based on MolVS [48]:

- **Disconnect Metals:** The MetalDisconnector is used to break bonds between metals and non-metal atoms, ensuring a consistent representation of metal coordination complexes.
- **Normalize the Molecule:** The molecule is normalized by adjusting bond orders and charges to produce a canonical form. Normalizing applies default SMARTS-based chemical transformations to reach normalized functional group representations.
- **Reionize the Molecule:** The Reionizer adjusts protonation states to ensure appropriate ionization. If this step fails, a warning is issued and the molecule proceeds without reionization.
- **Remove Charges:** The Uncharger attempts to remove formal charges from the molecule where chemically appropriate, resulting in a neutral representation.
- **Assign Stereochemistry:** Stereochemical Information is assigned to atoms and bonds when possible. Existing stereochemistry is preserved unless explicitly overridden.
- **Remove Atom Mapping Numbers:** Atom mapping numbers, commonly used in reaction mapping, are removed by resetting them to zero for all atoms.

### The Equality of Structures

A key consideration in structural comparison is the choice of format used to represent chemical structures. While InChI (International Chemical Identifier) is designed as a unique and standardized representation, SMILES is a widely used chemical line notation better applicable for cheminformatics applications [47]. Each format offers distinct advantages depending on the application [49].

The current InChI standard (version 1.06) has limitations in handling certain tautomeric forms, which may result in different tautomers being encoded as the same InChI [50]. Although SMILES can be reliably converted to InChI, the reverse transformation does not always yield consistent or unambiguous results with RDKit [23]. Furthermore, SMILES representations allow for explicit encoding of radicals, which is not supported completely by InChI [51]. Due to their conciseness, human readability [52, 53], and greater flexibility, MoleculeResolver by default returns structures as canonical isomeric SMILES.

Once the output format has been defined, the next challenge is determining structural equivalence. Multiple approaches to structure comparison exist, and selecting one involves balancing precision, performance, and use-case specificity. By default, MoleculeResolver compares canonical isomeric SMILES generated with RDKit [23] from standardized structures (see “Standardization and canonicalization” section) by character-wise string comparison. Resonance forms of ions are treated as equivalent, while tautomers and structures with differing isotopic composition are considered distinct.

This default behavior is fully configurable. Users can modify settings to specify whether tautomers, isomers, isotopes, or resonance structures should be treated as equivalent or distinct, as needed for a given application.

## The benchmark sets

**Table 2:** Benchmark datasets and corresponding identifiers

Dataset	Identifiers ($N_{components}$)	Description
SMILESRight	Name (6006), CAS RN® (5739)	Based on the corrected dataset by Glüge et al. [21], where multiple CAS RN® entries may refer to different isomers
CommChem	Name (35305), CAS RN® (35305)	Based on CAS Common Chemistry™ [28]. Names resolvable by OPSIN were removed, limiting the dataset to names retrievable via database search. Multiple names per structure are included
AERU	Name (1460)	Derived from a combination of PPDB [54], BPDB [55], and VSDB [56] from the Agriculture & Environment Research Unit [57]. Names resolvable by OPSIN were excluded
Electrolytes	Name (620)	A curated collection of mostly salts (ion combinations) and acids

Benchmarking the accuracy of identifier-to-structure resolution is challenging due to the absence of a universally accepted reference dataset. To address this, four benchmark datasets were constructed, mapping chemical names and CAS RN® to isomeric SMILES. Internal consistency checks were applied to all datasets. Entries lacking structural data, containing mixtures, or representing duplicates were removed. Only structures for which the isomeric and non-isomeric SMILES (if available) were in agreement were retained. This filtering process led to the exclusion of some entries.

Given the high reported precision of OPSIN (over 99.8%) [27], two of the benchmark datasets excluded names resolvable by OPSIN. This was done to evaluate the performance of MoleculeResolver in cases where rule-based structure generation is not feasible. The datasets were curated and evaluated between November 2024 and February 2025 and are available in the Supporting Information.

Three metrics are used in this work to evaluate the performance of MoleculeResolver under various configurations. These metrics are based on the number of true positives (TP), false positives (FP), and false negatives (FN):

Recall quantifies the proportion of correctly retrieved structures among all expected results:

1 $$\begin{aligned} \textbf{Recall} = \frac{TP}{TP + FN} \end{aligned}$$

Unless stated otherwise, TP includes only those structures that match the expected canonical isomeric SMILES.

Precision measures the proportion of correctly retrieved structures among all returned results:

2 $$\begin{aligned} \textbf{Precision} = \frac{TP}{TP + FP} \end{aligned}$$

The $\mathbf {F_1}$ score is the harmonic mean of precision and recall, providing a balanced assessment of performance:

3 $$\begin{aligned} \mathbf {F_1} = 2 \times \frac{\text {Precision} \times \text {Recall}}{\text {Precision} + \text {Recall}} \end{aligned}$$

## Results and discussion

### Crosschecking improves precision

**Fig. 2:**
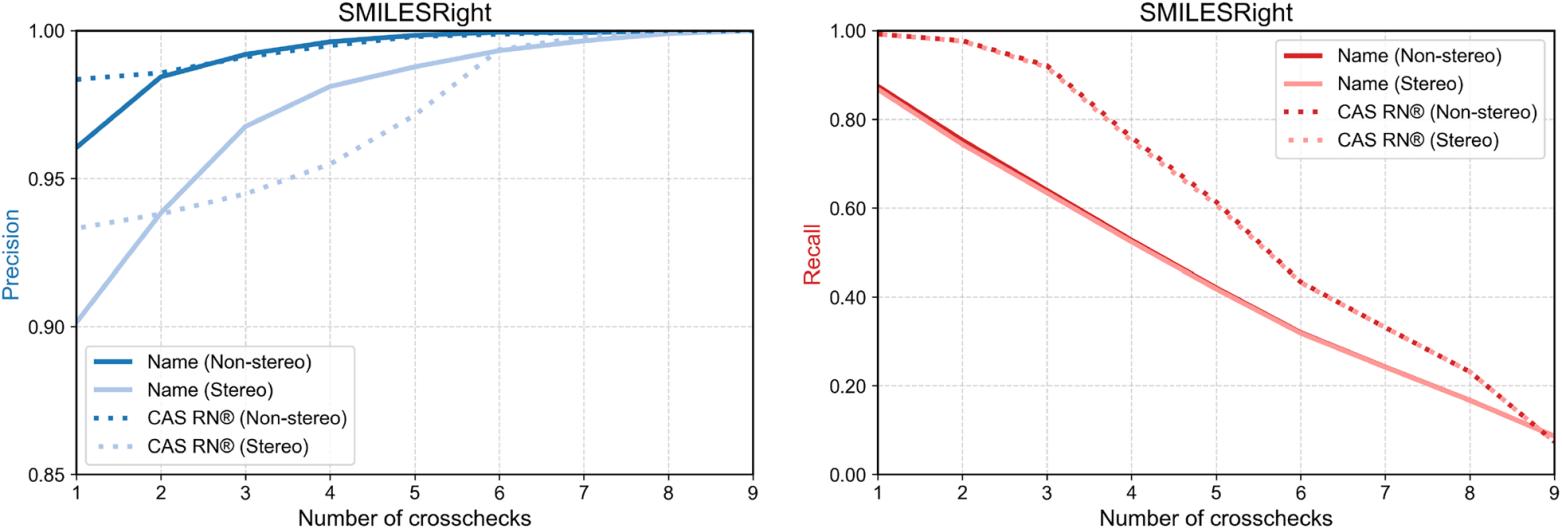
Precision and recall results for the SMILESRight dataset searched by name and CAS RN® as a function of the number of required crosschecks. The dark blue and dark red lines show the results considering components to be equal even if the structure did not match stereoisomeric information. The light blue and light red lines show the results considering components to be equal only if the structures also match stereoisomeric information

As shown in Fig. 2, applying multiple crosschecks across different services leads to improved precision. For both chemical names and CAS RN®, precision increases as the number of crosschecks rises. However, this improvement comes at the cost of reduced recall, as fewer components satisfy the minimum threshold for agreement. In the case of chemical names, recall decreases approximately linearly with the number of crosschecks. For CAS RN®, consistency across up to three databases is generally high.

This outcome is expected: chemical names are prone to minor variations—such as typographical errors or differences in naming conventions—that may prevent successful structure resolution. In contrast, CAS RN® can be validated algorithmically, making them more robust and less susceptible to such inconsistencies.

Whether or not to consider a structure to be correct if the stereoisomeric information matches depends on the usage case. In both cases studied, requiring the found structure to also match the stereoisomeric information of the reference database results in a precision reduction of approximately 5–6% when only one crosscheck is required. As the number of crosschecks increases, this difference becomes less pronounced.

Overall, the findings suggest that when a valid CAS RN® is available, it is generally more reliable to use it for structure retrieval rather than relying solely on chemical names. Nevertheless, querying both identifiers and comparing the resulting structures in cases of disagreement may offer the most robust approach, as it avoids assuming either identifier is inherently correct.

**Fig. 3:**
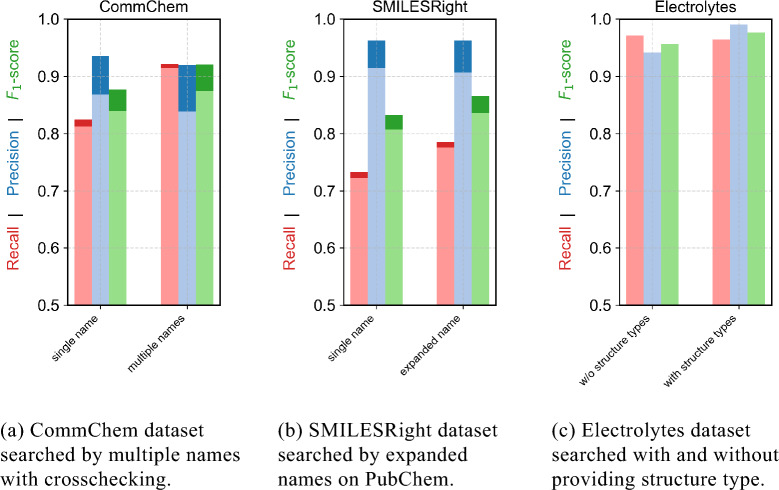
Results for the comparison of different name search methods. The dark blue, dark red and dark green show the results considering components to be equal even if the structure did not match stereoisomeric information. The light blue, light red and light green show the results considering components to be equal only if the structures also match stereoisomeric information. The Electrolytes benchmark set has almost no stereoisomeric information

### Searching for multiple names and heuristic name expansion improves $F_1$ score

MoleculeResolver supports querying multiple names for a single component, rather than relying on a single identifier. The algorithm sequentially searches all provided names in the given order across each service until one structure is found. Once a structure is retrieved from each service, the results are crosschecked. The CommChem dataset, which includes multiple names per component, was used to evaluate whether this approach improves performance. As shown in Fig. 3a, querying multiple names increases the overall $F_1$ score, with recall improving by more than 10%, while precision experiencing a slight decline.

In addition, MoleculeResolver includes a function for heuristic name expansion, which generates alternative name variants based on common errors observed in online databases. This heuristic was developed by empirical observations from processing large-scale datasets. The impact of this function varies, depending on the quality of the original names and the resolution capabilities of the target service. In some cases, it provides only marginal improvements over a single-name search (Figure 3b.

Whether heuristic name expansion will benefit a particular case can be assessed dynamically by checking whether the function returns additional name variants. This feature is particularly useful when resolving structures for ions or electrolytes. As illustrated in Fig. 3c, specifying the expected structure type (e.g., neutral compound, ion, salt, or mixture) can further improve results based on the $F_1$ score by up to 5%.

**Fig. 4:**
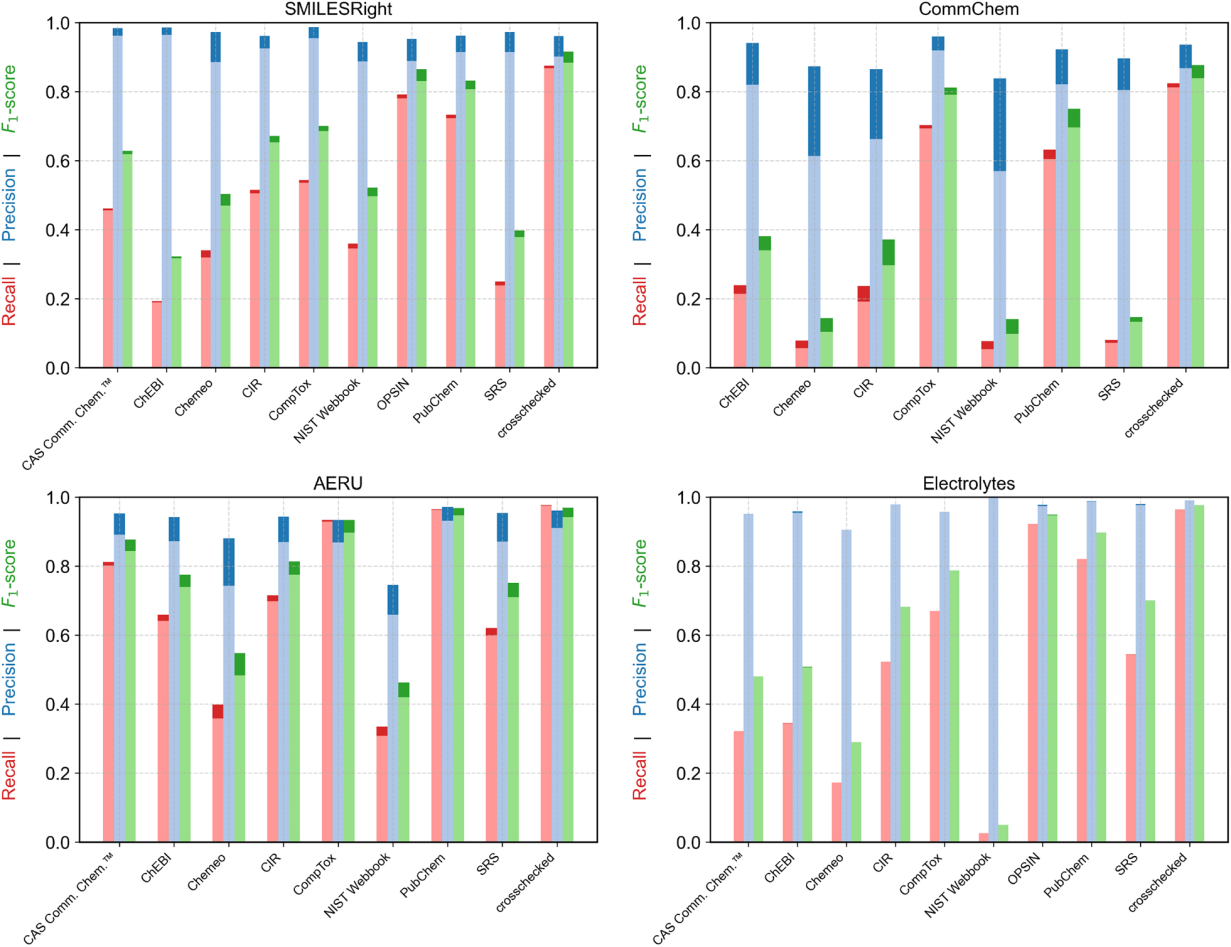
Results for searching the benchmark datasets by a single name with the different services and the crosschecked version with a number of crosschecks of one. The dark blue, dark red and dark green show the results considering components to be equal even if the structure did not match stereoisomeric information. The light blue, light red and light green show the results considering components to be equal only if the structures also match stereoisomeric information

### Overall performance on benchmark datasets

Searching for structures by name or CAS RN® represents a primary use case for MoleculeResolver. This has also been the main application in the author’s experience. Consequently, the module is evaluated specifically for these scenarios. In principle, reverse lookup is also supported—i.e., retrieving names or CAS RN® from other identifiers such as InChI, SMILES, or InChIKey.

It is worth noting that several studies [58–60] have discussed data exchange between CAS Common Chemistry™ [28], CompTox [33, 34], and PubChem [40], particularly with respect to synonyms and CAS RN®. As such, a degree of correlation among these services is expected. Nonetheless, they are treated as independent sources in this evaluation. As the results will demonstrate, internal post-processing workflows and differing search engines appear to yield distinct outputs.

#### Searching by name

In all cases, name-based searches using crosschecked results—i.e., those that aggregate structures from multiple services—achieve the highest $F_1$ scores (Fig. 4). Because no definitive benchmark exists, four separate datasets were employed in this study (cf. “The benchmark sets” section). Among these, the SMILESRight dataset, manually curated by Glüge et al. [21], likely provides the most accurate name-to-structure mappings. For this dataset, crosschecking yields the largest improvement over relying on any single service.

PubChem [40] is widely accessible and commonly used, in part due to its Python interface via PubChemPy [41]. As a result, many "independent" chemical structure repositories are likely influenced by the structures available in PubChem, a trend especially evident in the AERU dataset. As previously discussed, observable correlations may exist between CAS Common Chemistry™, CompTox, and PubChem. However, since MoleculeResolver utilizes the batch query functionality of both CompTox and PubChem, search operations remain efficient, and the benefit of higher accuracy through crosschecking offsets the modest increase in processing time.

#### Searching by CAS RN®

**Fig. 5:**
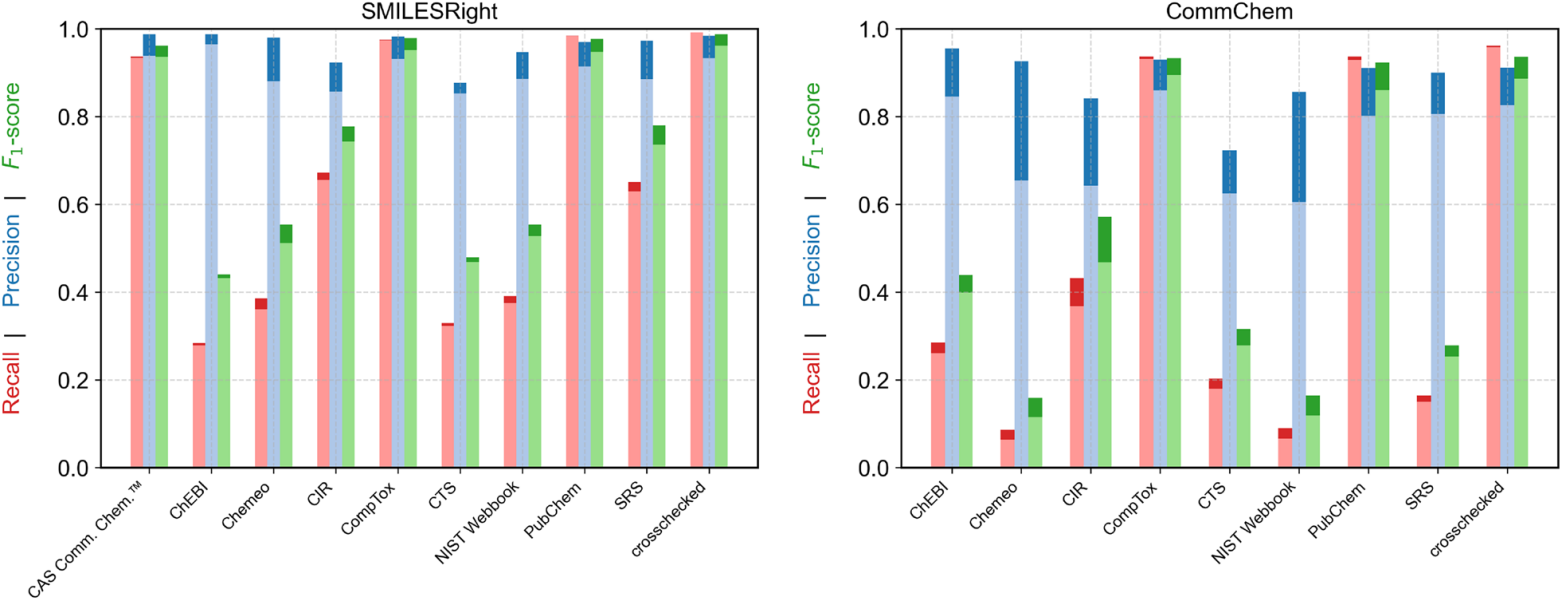
Results for searching the benchmark datasets by CAS RN® with the different services and the crosschecked version with a number of crosschecks of one. The dark blue, dark red and dark green show the results considering components to be equal even if the structure did not match stereoisomeric information. The light blue, light red and light green show the results considering components to be equal only if the structures also match stereoisomeric information

Similarly, for CAS RN®-based searches, the crosschecked approach consistently produces the highest $F_1$ scores (Fig. 5). In this case, agreement between CAS Common Chemistry™, CompTox, and PubChem is even stronger. Nonetheless, as seen with name-based searches, querying multiple services improves overall accuracy, despite a slight increase in runtime.

Overall, searches using CAS RN® tend to yield higher $F_1$ scores, with both recall and precision exceeding those of name-based queries. Therefore, when both identifiers are available and the CAS RN® is trusted to be correct, it is generally preferable to use it for structure retrieval.

### Performance on different combinations of services

**Fig. 6:**
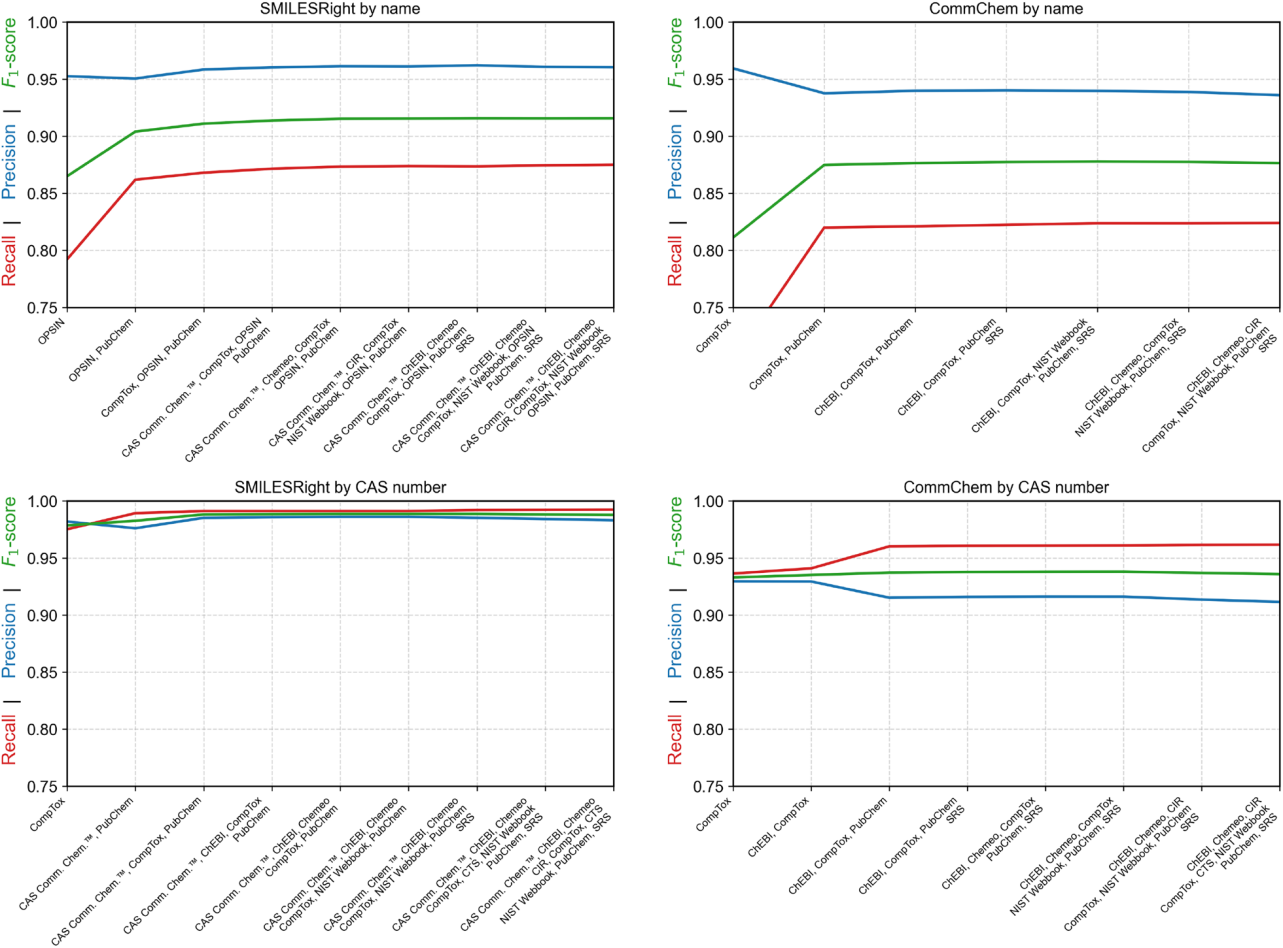
Results for searching the SMILESRight and CommChem datasets by different number of complementing services maximizing the $F_1$ score. Only results considering components to be equal even if the structure did not match stereoisomeric information are shown. The CommChem dataset excludes names resolvable by OPSIN

As a final evaluation, the performance of all possible $2^n$ subsets of services was systematically tested for different total number of services to determine which combinations yield the most accurate results. This analysis allows for the identification of complementary services that, when used together, improve overall structure resolution. Such insights can inform future configurations of MoleculeResolver, enabling users to select the most effective service subsets for a given identifier type.

For the SMILESRight dataset, which includes chemical names interpretable by OPSIN, OPSIN emerges as the most accurate individual service. As a rule-based name-to-structure converter, OPSIN’s performance highlights the value of algorithmic name resolution. Its inclusion in MoleculeResolver is further justified by the use of an offline batch version, which allows for highly efficient name resolution.

In contrast, the CommChem dataset excludes all names that can be resolved by OPSIN. As discussed earlier, this dataset reveals a strong correlation in performance among PubChem, CompTox, and CAS Common Chemistry™, reflecting shared data sources or synchronized updates.

Despite these overlaps, the results show that when resolving chemical names, OPSIN, CompTox, PubChem, and CAS Common Chemistry™ complement each other effectively across independently curated datasets. Their combination improves both recall and precision compared to any individual service.

When searching by CAS RN®, these same services—OPSIN excluded due to its focus on names—along with ChEBI, demonstrate strong complementarity. The combination of these services consistently outperforms individual sources, highlighting the benefits of multi-source crosschecking in resolving structured chemical identifiers (Fig. 6).

## Conclusion

This study presents MoleculeResolver as a practical and effective tool for automated chemical structure resolution, designed to address key challenges in mapping chemical identifiers to reliable canonical SMILES representations. By integrating multiple structure retrieval services, crosschecking algorithms, heuristic identifier expansion, and standardized molecular preprocessing, MoleculeResolver improves the consistency and reliability of structure assignment across diverse datasets.

Its modular and configurable architecture enables application across a range of use cases, from basic identifier resolution to more complex structure-based modeling tasks. Benchmarking against curated datasets demonstrated that MoleculeResolver outperforms individual services in terms of accuracy, particularly when dealing with ambiguous or error-prone identifiers. The combination of OPSIN’s rule-based parsing with database-driven retrieval enhances overall robustness and coverage.

In summary, MoleculeResolver facilitates more accurate and reproducible chemical data processing, helping to reduce errors introduced by manual or partially automated workflows. Its capabilities are likely to support improvements in data quality for computational chemistry, machine learning pipelines, cheminformatics, and regulatory or environmental modeling tasks.

## Data Availability

All datasets described in Table 2 are published on Zenodo: https://doi.org/10.5281/zenodo.15117784.
